# Antiprotozoal Activity of Turkish *Origanum onites* Essential Oil and Its Components

**DOI:** 10.3390/molecules24234421

**Published:** 2019-12-03

**Authors:** Deniz Tasdemir, Marcel Kaiser, Betül Demirci, Fatih Demirci, K. Hüsnü Can Baser

**Affiliations:** 1 Department of Pharmaceutical and Biological Chemistry, Centre for Pharmacognosy and Phytotherapy, School of Pharmacy, University of London, London WC1N 1AX, UK; 2 GEOMAR Centre for Marine Biotechnology, Research Unit Marine Natural Products Chemistry, GEOMAR Helmholtz Centre for Ocean Research Kiel, 24106 Kiel, Germany; 3 Kiel University, Christian-Albrechts-Platz 4, 24118 Kiel, Germany; 4 Department of Medical Parasitology and Infection Biology, Swiss Tropical and Public Health Institute, CH-4051 Basel, Switzerland; marcel.kaiser@swisstph.ch; 5 University of Basel, 4003 Basel, Switzerland; 6 Department of Pharmacognosy, Faculty of Pharmacy, Anadolu University, 26470 Eskisehir, Turkey; betuldemirci@gmail.com (B.D.); demircif@gmail.com (F.D.); khcbaser@gmail.com (K.H.C.B.); 7 Department of Pharmacognosy, Faculty of Pharmacy, Near East University, 99138 Nicosia, N. Cyprus

**Keywords:** *Origanum onites*, Lamiaceae, Turkish oregano, essential oil, carvacrol, thymol, antiprotozoal, *Trypanosoma*

## Abstract

Essential oil of *Origanum* species is well known for antimicrobial activity, but only a few have been evaluated in narrow spectrum antiprotozoal assays. Herein, we assessed the antiprotozoal potential of Turkish *Origanum onites* L. oil and its major constituents against a panel of parasitic protozoa. The essential oil was obtained by hydrodistillation from the dried herbal parts of *O. onites* and analyzed by Gas Chromatography-Flame Ionization Detector (GC-FID) and Gas Chromatography coupled with Mass Spectrometry (GC-MS). The in vitro activity of the oil and its major components were evaluated against *Trypanosoma brucei rhodesiense*, *T. cruzi*, *Leishmania donovani*, and *Plasmodium falciparum*. The main component of the oil was identified as carvacrol (70.6%), followed by linalool (9.7%), *p*-cymene (7%), γ-terpinene (2.1%), and thymol (1.8%). The oil showed significant in vitro activity against *T. b. rhodesiense* (IC_50_ 180 ng/mL), and moderate antileishmanial and antiplasmodial effects, without toxicity to mammalian cells. Carvacrol, thymol, and 10 additional abundant oil constituents were tested against the same panel; carvacrol and thymol retained the oil’s in vitro antiparasitic potency. In the *T. b. brucei* mouse model, thymol, but not carvacrol, extended the mean survival of animals. This study indicates the potential of the essential oil of *O. onites* and its constituents in the treatment of protozoal infections.

## 1. Introduction

The genus *Origanum* (Lamiaceae) is represented by 31 taxa including 27 species within the flora of Turkey [1]. Since ancient times, oregano has been used for flavoring fish, meat, vegetables, and wine, but also to treat various ailments in traditional Anatolian folk medicine. The first written records on the utilization of *Origanum* species in Anatolia date back to Hittite Empire times (1600–1200 BC) [2]. Leaves and inflorescences of various *Origanum* species (called “kekik” in Turkish) are still widely used as a condiment, herbal tea, for production of essential oil, and an aromatic water or hydrosol [3,4]. Orally, oregano oil preparations are used as a natural antispasmodic, analgesic, antimicrobial, expectorant, and carminative agent, and against coughs, digestive disorders, and menstrual problems. The oil is applied, topically, for its antiseptic and astringent effects, and widely consumed as an oral antiseptic and for gargling [4]. Oregano herb also serves as an important product for Turkish trade. Due to high quality product standards, Turkey has recently become a major and preferred supplier of *Origanum* herb and its oil to the world markets. The high demand for oregano and its essential oil has also fostered horticulture of *Origanum* sp. in the country, reducing wild harvest of the plant [5]. *Origanum onites* (Turkish oregano, Izmir oregano, Island oregano, or Cretan oregano) accounts for 80% of all the oregano exports of Turkey [6]. The plant is characterized by a relatively high yield of essential oil with high amounts (up to 80%) of carvacrol [4]. Similarly to other *Origanum* spp., the infusions and the essential oil of the aerial parts of *O. onites* are popular in treatment of gastrointestinal disorders, diabetes, high cholesterol, leukemia, bronchitis, and many other disorders in Turkish traditional medicine [5,7].

Vector-borne parasitic infectious diseases continue to threaten millions of people, particularly those living in third world countries. Malaria, caused by the protozoan parasite genus *Plasmodium*, is transmitted through female *Anopheles* mosquitoes. The intensive efforts of the WHO and non-profit organizations and increased public awareness in recent years halved the number of deaths due to malaria to 435,000 in 2017 [8]. However, other vector-borne diseases, such as trypanosomiasis or leishmaniasis, still remain neglected tropical diseases. *Trypanosoma brucei rhodesiense* is the etiological agent of human African trypanosomiasis (sleeping sickness, HAT) in eastern and southern Africa. The protist parasite enters the body through the bites of *Glossina* insects. Approximately 65 million people are at risk of HAT [9]. *Trypanosoma cruzi* causes Chagas’ disease (American trypanasomiasis), which is transmitted by blood-sucking triatomine bugs. About 7 million people, mostly in Latin America, are infected [10]. Leishmaniasis has three main forms—visceral, cutaneous, and mucocutaneous leishmaniasis—caused by different *Leishmania* parasites. Several *Leishmania* species (*L. donovani, L. infantum*, and *L. chagasi*) are the causative agents of visceral leishmaniasis (Kala-azar), which represents the most dangerous form of the disease. The parasite is transmitted to human by infected female phlebotomine sandflies. It is estimated that 50,000–90,000 new cases occur worldwide each year [11]. If left untreated, all these protozoal diseases are fatal. Although sustained efforts have reduced the numbers of cases and the death toll, the occurrence of drug-resistant strains and the adverse effects of the currently used drugs require new, safe, and effective antiprotozoal agents. 

As exemplified by *Cinchona* bark and *Artemisia annua* herba, as well as their active principles quinine and artemisinin, medicinal plants and their secondary metabolites represent highly valuable sources for new compounds against parasitic diseases. The majority of research efforts into the effects of plants on parasitic infections have used aqueous or alcoholic extracts, although essential oils are also efficacious in treating or preventing protozoal diseases [12]. Several *Origanum* spp. have been shown to inhibit the growth of internal and external parasites and protozoan infections in previous studies [13,14,15,16]. Turkish *Origanum onites* has also been extensively studied for numerous in vitro and in vivo biological activities [7,17]; however, no detailed study exists on its antiprotozoal activity. The aim of this present study was therefore to comprehensively evaluate the in vitro and in vivo antiprotozoal activity of Turkish *O. onites* essential oil and its 12 abundant components. 

## 2. Results

### 2.1. Chemical Composition of the Essential Oil

**Figure 1:**
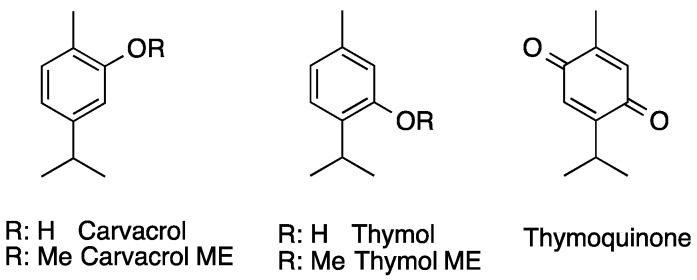
Structures of important essential oil components and their derivatives. ME: methyl ether.

Hydrodistillation of the aerial parts of *O. onites* delivered a relatively high yield of essential oil (2.0% v/w). Overall, 71 compounds, representing 99.9% of the total volatiles, were characterized by GC-FID and GC-MS analysis (Table 1). Chemically, the essential oil was predominated by carvacrol (70.6%, Figure 1). The other major constituents of the oil were linalool (9.7%), *p*-cymene (7.0%), γ-terpinene (2.1%), and thymol (1.8%). Reasonable levels of myrcene and α-terpinene (each 1%), terpinen-4-ol, β-caryophyllene, and α-terpineol (all 0.7%), as well as α-pinene and borneol (both 0.5%), were also detected. Oxygenated monoterpenes comprised the largest portion of the oil composition (84.9%), but monoterpene hydrocarbons (12.9%), sesquiterpene hydrocarbons (1.1%), and other components (13%) e.g., alcohols, esters, and aldehydes were also determined in the oil. 

### 2.2. In Vitro Antiprotozoal Activity and Cytotoxicity of the Essential Oil

The essential oil was tested in vitro for activity against blood-stage forms of multidrug-resistant *Plasmodium falciparum,* trypomastigote forms (mammalian stage) *of Trypanosoma brucei rhodesiense* and *T. cruzi*, and towards amastigotes (the clinically relevant form) of *Leishmania donovani* parasites. As shown in Table 2, the oil showed significant in vitro activity against African trypanosome *T. b. rhodesiense* (IC_50_ 0.18 μg/mL) and moderate antileishmanial (IC_50_ 17.8 μg/mL) and antiplasmodial (IC_50_ 7.9 μg/mL) effects. No inhibitory activity was observed against the American trypanosome *T. cruzi* at the highest concentration tested (90 μg/mL). The oil was devoid of any toxicity against the mammalian cell line (L6) even at the highest test concentration (>90 μg/mL).

### 2.3. In Vitro Activity and Cytotoxicity of the Individual Constituents of the Essential Oil

The significant in vitro antiprotozoal activity of the essential oil encouraged us to test carvacrol, the main phenolic monoterpenoid constituent of the oil, as well as its position isomer, thymol (Figure 1). Interestingly, both compounds exhibited almost identical antitrypanosomal activities against *T. b. rhodesiense*, with IC_50_ values of 110 ng/mL (thymol) and 150 ng/mL (carvacrol) (Table 2). Their leishmanicidal and plasmocidal potentials were also highly comparable to that of the oil, and both compounds were inactive against *T. cruzi* (Table 2). Pure carvacrol and thymol showed some low toxicity against mammalian cells; however, their selectivity indices (IC_50_ value against L6 mammalian cells/IC_50_ value against parasite), for example against *T. b. rhodesiense*, were very high (454.4 for thymol and 327.5 for carvacrol, Table 2). 

The significant antiprotozoal activity of carvacrol and thymol prompted us to test their derivatives, carvacrol methyl ether and thymol methyl ether (Figure 1). The latter compound (thymol ME) showed moderate antitrypanosomal activity against African trypanosomes (IC_50_ 4.1 μg/mL), and this was four times stronger than that observed for CME (IC_50_ 17.0 μg/mL). However, both activities were significantly lower than the parent compounds (Table 2). We further tested thymoquinone (Figure 1), the quinone derivative of thymol. This compound was equipotent towards *T. b. rhodesiense*, with identical IC_50_ values to thymol (IC_50_ 110 ng/mL), and showed approximately 3- to 10-fold higher activities against *L. donovani* (IC_50_ 1.7 μg/mL) and *P. falciparum* (IC_50_ 2.6 μg/mL). However, during the first round of antiprotozoal testing, we observed instability of the thymoquinone sample, which exhibited a color change. When the testing was repeated (four times) against the most susceptible parasite, *T. b. rhodesiense*, the observed IC_50_ values increased severely from 0.123 μg/mL to 039 μg/mL, 8.61 μg/mL, and finally to 42.1 μg/mL. In initial testing, thymoquinone exerted 2.5-fold higher cytotoxicity on L6 cells (IC_50_ 20.5 μg/mL) than thymol (Table 2), indicating a lower selectivity towards parasites.

Carvacrol is the main component of the essential oil (70.6%), whereas thymol, which exists therein with much lower abundance (1.8%), exhibited in vitro antiparasitic activity equal to carvacrol. This prompted us to test other major constituents of the oil, i.e., *L*-linalool (9.7%), p-cymene (7.0%), and γ-terpinene (2.1%), plus several other components of the oil. For the latter, with comparatively lower percentages in the oil, we set a threshold (0.5%) to evaluate their potential. Thus, in addition to carvacrol and thymol, 10 additional constituents of the oil were also submitted to the same in vitro bioactivity assessments. As displayed in Table 2, only α-pinene, α-terpineol, and terpinen-4-ol showed remarkable trypanocidal activity towards *T. b. rhodesiense* (IC_50_ values 0.42, 0.56, and 0.66 μg/mL, respectively) and displayed low or moderate toxicity to L6 cells (IC_50_ values 87.8, 32.3, and 43.3 μg/mL, respectively). In addition, α-terpinene showed equipotent trypanocidal (*T. b. rhodesiense*) and antimalarial potential (IC_50_ values 3.1 and 3.7 μg/mL, respectively). *L*-Linalool was the last compound with reasonable antitrypanosomal activity against *T. b. rhodesiense* (IC_50_ 3.6 μg/mL). All other compounds showed low to no in vitro antiprotozoal activity against the set of protozoan parasites in the test panel (Table 2).

### 2.4. In Vivo Trypanocidal Activity of Carvacrol and Thymol

Based on their potent trypanocidal activities, the essential oil of *O. onites* and its main constituent carvacrol were selected for an in vivo activity study against *T. brucei brucei* STIB795 mouse model. We also decided to test thymol, which exhibited equal in vitro activity to carvacrol, on the same animal model for comparison. The oil and its components were applied intraperitoneal (i.p) at a 100 mg/kg dose, but failed to show any significant in vivo activity. Notably, the mice treated with thymol showed a mean survival of 9 days, which represented an extension of survival compared to 5.25 days for the untreated control group (Table 3). The mice treated with the positive control (pentamidine) were cured and survived over 30 days. Due to the limited in vivo efficacy of the tested compounds, other less active compounds, namely α-pinene, α-terpineol, and terpinen-4-ol, were not submitted to an in vivo study. Thymoquinone, which was equally active against *T. b. rhodesiense* in vitro to carvacrol and thymol, was also excluded from animal studies based on its relatively higher in vitro toxicity and instability observed during the in vitro testing.

## 3. Discussion

Similarly to many Lamiaceae plants, *Origanum* species accumulate high levels of essential oil with varying yield and chemical composition, depending on location or origin. *O. onites* is a narrowly distributed East Mediterranean species growing mainly in Turkey and Greece, which comprises one of the major herbs of the oregano trade worldwide [18]. In a previous study, the essential oil of Greek *O. onites* collected from Chios, a Greek island bordering with the Turkish mainland, gave the highest essential oil yield (3%–7% v/m dry leaves and inflorescences) and the highest carvacrol content (69.0%–92.6%) ([18]. Turkish *O. onites* has two chemotypes: (i) carvacrol-type (with 66.5%–80.4% carvacrol content) and (ii) linalool-type (with 90.0%–91.9%) linalool content) [6]. Obviously, the plant material studied herein represents a carvacrol chemotype. The oil yield and the chemical composition of the Turkish *O. onites* essential oil also show seasonal variations. The maximum essential oil and highest carvacrol content in the leaves has been demonstrated to occur in July [19].

In Turkey, *O. onites* herb and its essential oil and hydrosol are routinely used for a number of ailments, and have been tested for various biological and pharmacological activities [7]. In the continuation of our efforts to identify antiprotozoal plants and natural products from Turkish Lamiaceae plants [20,21,22], we decided to study the in vitro antiprotozoal activity of Turkish *O. onites* essential oil against a panel of protozoan parasites, and tested its potential toxicity towards a mammalian cell line to determine the selectivity. The oil was moderately active against *P. falciparum* and *L. donovani*, but exhibited very high efficacy against trypanomastigote forms of African trypanosome *T. b. rhodesiense*, while being inactive versus the same forms of American trypanosome *T. cruzi*. Furthermore, the oil showed no toxicity towards mammalian cells. Thus, we decided to assess the in vitro activity of carvacrol, the principle compound of the *O. onites* oil, its isomer thymol, and 10 other individual terpenoid components (with up to 0.5% abundance in the oil) for their potential contribution to the overall activity of the oil. Carvacrol and thymol exerted activity equal to that of the oil against all test organisms, with a very high selectivity to *T. b. rhodesiense*. When tested for in vivo trypanocidal activity in mice, only thymol extended the survival time of infected mice, whereas the essential oil and carvacrol were inactive. To our knowledge, this is the first report of any in vivo activity of thymol in a *T. brucei* in vivo animal model.

The antimicrobial activity of essential oils and their components has long been recognized, and has continued to receive research interest in recent years. In comparison to numerous antibacterial or antifungal activity screening studies, little work has been done to assess the potential of volatile oils and their individual constituents for treatment of endo- or ectoparasites [12,23]. Essential oils of a few *Origanum* species have been reported to exhibit antiparasitic activities against human or animal parasites. In human trials, orally given emulsified oregano oil has shown high activity against intestinal parasites *Blastocystis hominis, Entamoeba hartmanni*, and *Endolimax nana* [14]. A powder containing 5% Greek *O. vulgare subsp. hirtum* oil (and 95% natural feed-grade inert carrier) was also effective against chickens infected with *Eimeria tenella*, an endoparasite that causes hemorrhagic cecal coccidiosis in poultry [15]. A highly diluted (1%) aqueous solution of oregano oil kills head lice, *Pediculus humas capitis*, an ectoparasite [13].

The essential oils of several *Origanum* species have been submitted to narrow-spectrum antiprotozoal activity studies, generally on single parasites or different species of the same parasite genus. Fujisaki and coworkers (2012) evaluated the antiplasmodial activity of 47 essential oils, including oregano oil (species name of the plant and full chemical composition of the oil were not given) containing 26% carvacrol. The oil, as well as its constituents thymol and carvacrol, showed in vitro antimalarial activity against *P. falciparium* FCR-3 strain with an IC_50_ value of 10 μg/mL [24]. In vitro antimalarial potency of the essential oil of *O. vulgare* [25] and *O. compactum* collected from Morocco [26] ranged from very low to moderate, respectively. Carvacrol was reported to be inactive against chloroquine-resistant *P. falciparum* strain (IC_50_ > 1 mM) [27], whereas thymol exhibited in vitro antimalarial activity with IC_50_ value of 4.5 μg/mL [28]. The latter activity is very similar to that observed in our study.

Brazilian *O. virens* essential oil showed little activity against *L. infantum* promastigotes (IC_50_ 196 μg/mL, MTT assay 24 h) [29], and even poorer activity was exerted by Brazilian *O. vulgare* towards *L. amazonesis* promastigotes (IC_50_ 405.5 μg/mL, MTT, 72 h), with the major components cis-*p*-menth-2-en-1-ol (33.9%) and linalyl acetate (13.9%) [30]. When the essential oil from the flowering stage aerial parts of Moroccan *O. compactum* was tested against *L. tropica, L. major*, and *L. infantum*, the highest potency was observed against the latter (IC_50_ 0.02 μg/mL, MTT, 72 h incubation). This oil contains comparable amounts of carvacrol, thymol, *p*-cymene, and γ-terpinene [31]). Moderate in vitro activity of carvacrol against various *Leishmania* spp., for example against *L. infantum* (IC_50_ value of 7.35 μg/mL), has been demonstrated [32]. Monoterpene phenols thymol and carvacrol show activity against promastigote forms of *L. chagasi* [33,34] with IC_50_ values of 9.8 μg/mL (thymol) and 2.3 μg/mL (carvacrol). Semi-synthetically prepared Morita–Baylis–Hillman adducts of thymol and carvacrol exert superior activity and lower toxicity in vitro than their parent compounds against promastigotes of *L. amazonensis*, one of the causative agents of cutaneous leishmaniasis [35]. Thymol, as well as its benzoyl and acetyl derivatives, exhibit in vitro leishmanicidal activity against both promastigotes and amastigotes of *L. infantum chagasi* [36]. A very recent study by Youssefi et al. (2019) reported the moderate and comparable leishmanicidal effects of both carvacrol and thymol against *L. infantum* promastigotes (IC_50_ values 9.8 and 7.2 μg/mL, respectively) upon a 24 h MTT assay [37]. Several *Origanum* spp. essential oils, as well as thymol and carvacrol, have been evaluated against several *Leishmania* species before, but the current study reported for the first time the effect of oregano oil and its constituents on amastigotes (the clinically relevant form) of *L. donovani* causing visceral leishmaniasis, the most serious form of the disease.

Santoro et al. (2007) investigated the effect of essential oil of *O. vulgare* on the growth and ultrastructure of evolutionary forms of American trypanosome *T. cruzi* [38]. The IC_50_ values obtained by 24 h incubation of epimastigotes and trypomastigotes with oregano oil were low (175 and 115 μg/mL, respectively). Thymol, tested at the same test conditions, showed a better efficacy, with IC_50_ values of 62 μg/mL (epimastigotes) and 53 μg/mL (trypomastigotes) [38]. In another study by Escobar et al. (2010), thymol (IC_50_ 3.2 μg/mL) was found to be active against intracellular amastigotes of *T.-cruzi*-infected Vero cells, with a selective index greater than 10 [39].

Human African trypanosomiasis is caused by two subspecies of *T. brucei brucei*, namely *T. brucei gambiense* and *T. brucei rhodesiense*. The essential oil of Portuguese *O. virens*, with a somewhat similar chemical composition (carvacrol 68.2%, γ-terpinene 7.9%, *p*-cymene 7.4%, β-myrcene 2.4%, and thymol 2.1%) to Turkish *O. onites* showed moderate in vitro trypanocidal activity against *T. b. brucei* (IC_50_ 24.0 μg/mL) [40]. These results are 130 times lower than what we obtained with *O. onites* oil against *T. brucei rhodesiense*. The discrepancy may stem from the use of different *Trypanosoma* species (AnTart 1 strain, wild type), incubation time, and different methodologies. Carvacrol and thymol demonstrated activity against *T. brucei brucei* (life stage or form has not been reported) with IC_50_ values of 11.3 and 22.9 μg/mL [41]. To our knowledge, thymol has never been tested or reported to have in vitro activity against *T. brucei rhodesiense* subspecies.

Several studies have assessed the in vivo activity of thymol and its semi-synthetic derivatives against *Leishmania-* and *Trypanosoma*-type parasitic kinetoplastids. The administration of benzoyl and acetyl–thymol to BALB/c mice infected with *L. infantum chagasi* at 100 mg/kg/day (i.p.) dose for 30 days demonstrated in vivo activity [36]. Despite the low leishmanicidal activity, the oral administration of thymol (and its di-nitro derivative) in mice for 30 days reduced the parasite burden significantly (67.8% versus the standard glucantime 100%) [42]. Thymol was also shown to exhibit anti-*Trypanosoma-cruzi* effect in vivo. Upon application of thymol to infected albino mice at 200 mg/kg/day dose by intragastric route, a significant reduction in parasitemia on the 22nd day post-infection was observed [43]. Youssefi et al. (2019) tested carvacrol, thymol, and linalool (100 mg/kg, i.p. route) in hamsters infected with *L. infantum*, and concluded thymol to be the most effective and the safest metabolite with lowest side effects on the hamster liver [37]. To our knowledge, ours was the first in vivo study showing the potential of thymol to extend the life span of mice infected with African trypanosomes in a *T. brucei brucei* model.

Among the other three major constituents of the Turkish *O. onites* essential oil (linalool, *p*-cymene, and γ-terpinene), only *L*-linalool, which is the main linalool enantiomer found in *Origanum* essential oils [44], showed reasonable *T. b. rhodesiense* activity (IC_50_ 3.6 μg/mL). *p*-Cymene and γ-terpinene were devoid of any significant activity against any of the parasites tested. When the other seven constituents with up to 0.5% concentrations in the oil were evaluated against the protozoan panel, *T. b. rhodesiense* emerged as the most susceptible parasite. In particular, α-pinene, terpinen-4-ol, and α-terpineol were most active, however they lacked significant activity to other three parasites in the test panel (Table 2). Mikus et al. (2000) have shown good activity of terpinen-4-ol towards *T. brucei* bloodstream forms (ED_50_ 0.02 μg/mL) with 1000-fold more selectivity to the parasite than to the human cell line [45]. α-Pinene has been shown to be active against *T. cruzi* epimastigotes and amastigotes (IC_50_ values 2.74 and 1.92 μg/mL, respectively [46], as well as blood stream trypomastigotes cultures of *T. brucei brucei* with an IC_50_ value of 2.9 μg/mL [40]. To our knowledge, this study was the first report of α-terpineol. α-Terpinene was equipotent against both *T. b. rhodesiense* and *P. falciparum* with negligible toxicity. Treatment of *T. evansi*-infected mice with α-terpinene has been shown to extend animal longevity [47]. The current study reported for the first time in vitro antiplasmodial, antileishmanial. and the trypanocidal activity against *T. b. rhodesiense* and *T. cruzi* of α-terpinene.

The mechanism underlying the antimicrobial, including antiprotozoal, actions of essential oils and their monoterpenes is not fully understood [34]. Due to their highly lipophilic nature, which permits easy absorption by the cell membrane, it was generally accepted that they were involved in the alteration and disruption of lipophilic membranes and inhibition of parasites’ lipid metabolism [48,49]. However, a flow cytometry study performed by Santoro et al. (2007) indicated that the essential oils permeate the cell membrane and kill the parasites by affecting cytoplasmic metabolic pathways or organelles, and not by compromising the parasite membrane integrity [38]. Therefore, it seems the mechanism of action for oils and their constituents is rather complex. Several new studies investigated the potential targets using in silico methods [50,51].

The current study showed that carvacrol and thymol fully retain the in vitro antiprotozoal activity of the *O. onites* essential oil, without showing significantly superior activity to the oil. Such a phenomenon, i.e., that the single main components of essential oils do not exceed the activity of the original oil, has been observed previously [52]. Some studies have shown a synergistic antiparasitic activity, in vitro and in vivo, of a combination of several single oil components [52,53]. It is also noteworthy that thymol and carvacrol were equipotent in vitro against *T. b. rhodesiense*. When tested in vivo in mice, thymol had some effect, but carvacrol and the essential oil were inactive. Discrepancy between in vitro and in vivo efficacy is a complex and common issue in drug discovery stemming from, for example, low solubility, in vivo instability, hydrolysis, and oxidation of the compound of interest. Essential oils are oily mixtures of lipophilic, liquid, and volatile compounds. Diminished in vivo effects of essential oils in animals have been reported [54,55]. Such non-optimal pharmacokinetic and physiochemical properties, for example, very rapid absorption, poor solubility, poor bioavailability, and elimination rate, are considered as major hurdles in the development of essential oils and their volatile components as drugs [56,57]. Solid formulation techniques (e.g., microencapsulation, liposomes, nanoparticles) are used for their controlled release and targeted delivery of such hydrophobic oils [57,58,59,60]. Such techniques also address additional issues related to essential oils and their terpenoid constituents (strong odor, taste, evaporation, degradation, and sensitivity to light, air, and temperature) [61].

The importance of the presence of the hydroxyl group in the bioactivity of phenolic compounds such as carvacrol and thymol is well known [62,63]. Accordingly, the methyl ether derivatives of these compounds were 40–90 times less potent towards *T. b. rhodesiense* than the original compounds (Table 2). The activity of thymol is attributed to the characteristic feature of the phenolic hydroxyl group, which may be more acidic than carvacrol, and hence more active, due to the presence of a system of delocalized electrons [63]. Thymoquinone has equipotent trypanocidal activity to thymol, and exerts enhanced antileishmanial and antiplasmodial effects; however, due to observed instability during in vitro assays, it was excluded from the animal model studies. To our knowledge, this is most comprehensive and in-depth study performed on the essential oil of any *Origanum* species that included in vitro antiprotozoal activity assessments of the oil and its 12 constituents against four different parasitic protozoa, cytotoxicity, and finally in vivo activity testing.

## 4. Materials and Methods

### 4.1. Reagents and Chemicals

Pure essential oil compounds in high purity (>95%), reagents, and solvents were obtained from various commercial sources, i.e., Sigma-Aldrich, Fluka, Merck, Frutarom, and Roth. The origin of the standard compounds used in bioassays are as follows: chloroquine diphosphate (Sigma-Aldrich, Buchs, Switzerland), benznidazole (Epichem Ltd., received from DNDi, Geneva), melarsoprol (Arsobal Sanofi-Aventis, received from WHO, Geneva), miltefosine (Sigma-Aldrich, Buchs, Switzerland), podophyllotoxin (Sigma-Aldrich, Buchs, Switzerland), and pentamidine (Sigma-Aldrich, Buchs, Switzerland).

### 4.2. Plant Material and Isolation of the Essential Oil

The plant material was acquired from Altes Ltd. (Antalya, Turkey) and identified by one of us (K.H.C. Baser, Turkey). A voucher specimen is deposited at Department of Pharmacognosy, Faculty of Pharmacy, Anadolu University, Eskisehir (ESSE 14567). The whole air-dried aerial parts of the plant were subjected to hydrodistillation for 3 h using a Clevenger apparatus to produce the essential oil. The oil yield was 2% (*v*/*w*).

### 4.3. Gas Chromatography Analyses

The *O. onites* oil was analyzed by GC-FID and GC-MS using an Agilent GC–mass selective detector (MSD) system. The GC-MS analyses were carried out with an Agilent 5975 GC-MSD system. An Innowax fused silica capillary column (60 m × 0.25 mm, 0.25 μm film thickness) was used with helium as the carrier gas (0.8 mL/min). The oven temperature was kept at 60 °C for 10 min, then programmed to 220 °C at a rate of 4 °C/min, then maintained constant at 220 °C for 10 min, and finally programmed to 240 °C at a rate of 1 °C/min. The injector temperature was set at 250 °C. The split flow was adjusted at 50:1. Mass spectra were recorded at 70 eV over the mass range m/z 35–450. The GC analyses were performed using an Agilent 6890N GC system. The FID detector temperature was set to 300 °C, and the same operational conditions were used with a duplicate of the same column employed in GC-MS analyses. Simultaneous autoinjection was done to obtain equivalent retention times. Relative percentages of the separated compounds were calculated from integration of the peak areas in the GC-FID chromatograms (Table 1). Identifications of the essential oil components were carried out by comparison of their relative retention times with those of authentic samples or by comparison of their relative retention indexes (RRI) to series of *n*-alkanes. Computer matching against commercial (Wiley GC/MS Library, MassFinder Software 4.0) [64,65] and in-house “Başer Library of Essential Oil Constituents” was built up by genuine compounds and components of known oils.

### 4.4. In Vitro Assay for Plasmodium falciparum

Activity against the erythrocytic stages of *P. falciparum* was determined using an in vitro modified [^3^H]-hypoxanthine incorporation assay [66,67], using the chloroquine- and pyrimethamine-resistant K1 strain [68]. Compounds were dissolved in DMSO at 10 mg/mL and further diluted in medium before added to parasite cultures incubated in RPMI 1640 medium without hypoxanthine, supplemented with HEPES (5.94 g/l), NaHCO_3_ (2.1 g/L), neomycin (100 U/mL), AlbumaxR (5 g/L), and washed human red cells A+ at 2.5% haematocrit (0.3% parasitaemia). Serial drug dilutions of seven 3-fold dilution steps covering a range from 90 to 0.123 μg/mL were prepared. The 96 well plates were incubated in a humidified atmosphere at 37 °C; 4% CO_2_, 3% O_2_, 93% N_2_. After 48 h 50 μL of ^3^H-hypoxanthine (=0.5 μCi) was added to each well of the plate. The plates were incubated for a further 24 h under the same conditions. The plates were then harvested with a Betaplate™ cell harvester (Wallac, Zurich, Switzerland), and the red blood cells transferred onto a glass fiber filter then washed with distilled water. The dried filters were inserted into a plastic foil with 10 mL of scintillation fluid, and counted in a Betaplate™ liquid scintillation counter (Wallac, Zurich, Switzerland). The results were recorded as counts per minute (CPM) per well at each drug concentration and expressed as percentage of the untreated controls. IC_50_ values were calculated from the sigmoidal inhibition curves using Microsoft Excel. Chloroquine was used as positive control drug.

### 4.5. In Vitro Assay for Trypanosoma brucei rhodesiense

*Trypanosoma brucei rhodesiense* STIB 900 strain, isolated in 1982 from a human patient in Tanzania and, after several mouse passages, cloned and adapted to axenic culture conditions, was used in this assay [69]. Minimum essential medium (50  μL) supplemented with 25  mM HEPES, 1  g/L additional glucose, 1% MEM non-essential amino acids (100×), 0.2  mM 2-mercaptoethanol, 1  mM Na-pyruvate, and 15% heat inactivated horse serum was added to each well of a 96 well microtiter plate. Serial drug dilutions of seven 3-fold dilution steps covering a range from 90 to 0.123  μg/mL were prepared, and then 4 × 10^4^ blood stream forms of *T. b. rhodesiense* STIB 900 in 50  μL were added to each well and the plate was incubated at 37  °C under a 5% CO_2_ atmosphere for 72  h. Resazurin solution (resazurin, 12.5  mg in 100  mL double-distilled water) (10  μL) was then added to each well and incubated for a further 2–4  h [70]. At the end of the assay, the plates were read with a Spectramax Gemini XS microplate fluorometer (Molecular Devices Cooperation, Sunnyvale, CA, USA) using an excitation wavelength of 536  nm and an emission wavelength of 588  nm. Data were analyzed using the microplate reader software Softmax Pro (Molecular Devices Cooperation, Sunnyvale, CA, USA). Melarsoprol was used as standard drug.

### 4.6. In Vitro Assay for Trypanosoma cruzi

Rat skeletal myoblasts (L-6 cells) were seeded in 96 well microtiter plates (2000 cells/well) in 100  μL RPMI 1640 medium with 10% FBS and 2  mM L-glutamine for 24  h. After that, the medium was removed and replaced by 100  μL per well containing 5000 trypomastigote forms of *T. cruzi* Tulahuen strain C2C4, containing the β-galactosidase (*Lac Z*) gene [71]. The medium was removed from the wells after 48 h, and replaced with 100 μL fresh medium with or without a serial drug dilution of seven 3-fold dilution steps covering a range from 90 to 0.123 μg/mL. After 96 h of incubation, the plates were inspected under an inverted microscope to assure growth of the controls and sterility, after which the substrate CPRG/Nonidet (50 L) was added to all wells. A color reaction developed within 2–6 h and could be read photometrically at 540 nm. Data were transferred into the graphic program Softmax Pro (Molecular Devices Cooperation, Sunnyvale, CA, USA) and the IC_50_ values were calculated from the sigmoidal dose response curves. Benznidazole was the reference drug.

### 4.7. In Vitro Assay for Leishmania donovani

Amastigotes of *L. donovani* (strain MHOM/ET/67/L82) were grown in axenic culture at 37  °C in SM medium [72] at pH 5.4 supplemented with 10% heat-inactivated FBS under an atmosphere of 5% CO_2_. Culture medium (100 μL) with 10^5^ amastigotes from axenic culture with or without a serial drug dilution was seeded in 96 well microtiter plates. Serial drug was prepared covering a range of dilutions from 90 to 0.123 μg/mL. After 72 h of incubation, the plates were inspected under an inverted microscope to assure growth of the controls and sterile conditions. Subsequently, 10 μL resazurin solution (12.5 mg resazurin dissolved in 100 mL double-distilled water) was added to each well and the plates were incubated for another 2 h. The plates were then read with a Spectramax Gemini XS microplate fluorometer (Molecular Devices Cooperation, Sunnyvale, CA, USA) using an excitation wavelength of 536 nm and an emission wavelength of 588 nm. Data were analyzed using the software Softmax Pro (Molecular Devices Cooperation, Sunnyvale, CA, USA). A decrease of fluorescence (=inhibition) was expressed as percentage of the fluorescence of control cultures and plotted against the drug concentrations. From the sigmoidal inhibition curves, the IC_50_ values were calculated. Miltefosine was used as a reference drug.

### 4.8. In Vitro Assay for Cytotoxicity on Mammalian Cells

Assays were performed in 96 well microtiter plates (4  ×  10^4^  L6 cells/well), each well containing 100  μL of RPMI 1640 medium supplemented with 1% L-glutamine (200  mM) and 10% FBS. L6 cells are a primary cell line derived from rat skeletal myoblasts [73,74]. Serial drug dilutions of seven 3-fold dilution steps covering a range from 90 to 0.123 μg/mL were prepared. After 72 h of incubation, the plates were inspected under an inverted microscope to assure growth of the controls and sterile conditions. Resazurin solution (10 μL) (12.5 mg resazurin in 100 mL double-distilled water) was then added to each well and the plates incubated for another 2 h. The plates were then read with a Spectramax Gemini XS microplate fluorometer (Molecular Devices Cooperation, Sunnyvale, CA, USA) using an excitation wavelength of 536 nm and an emission wavelength of 588 nm. Data were analyzed using the microplate reader software Softmax Pro (Molecular Devices Cooperation, Sunnyvale, CA, USA). From the sigmoidal inhibition curves, the IC_50_ values were calculated. Podophyllotoxin was the positive control.

### 4.9. In Vivo Trypanocidal Activity Assessment

In vivo efficacy studies in mice were conducted at the Swiss Tropical and Public Health Institute (Basel) (License number 2813) according to the rules and regulations for the protection of animal rights (“Tierschutzverordnung”) of the Swiss “Bundesamt für Veterinärwesen”. They were approved by the veterinary office of Canton Basel-Stadt, Switzerland. Groups of four female NMRI mice weighing 20 to 25 g were infected i.p. on Day 0 (d0) with 10^5^ bloodstream forms of *T. brucei brucei* STIB 795, which is a derivative of strain 427. Compounds were formulated in 100% DMSO diluted 10-fold in distilled water. Mice were treated on four consecutive days (d3 to d6 post-infection) with the *Origanum onites* oil, as well as carvacrol and thymol, at 100 mg/kg by the i.p. route. One group served as untreated controls, and two other groups were treated with the standard drugs pentamidine (5 mg/kg for 4 days) and melarsoprol (2 mg/kg for 4 days), respectively. The levels of parasitemia of the mice were checked by microscopic examination of tail blood on Day 7 and thereafter twice a week until Day 30 post infection. Mice were considered cured when there was no parasitaemia relapse detected in the tail blood over the 30 day observation period. The day of relapse and surviving days were recorded.

## 5. Conclusions

Oregano oil is known to show a wide range of health benefits. The current study added another potential use of oregano oil and its constituents in the area of infectious parasitic diseases. Although the oil and its main constituents lack significant in vivo curative activity in mice, they may have synergistic effects and exhibit enhanced bioactivity when combined with other constituents of the oil, or with clinically used antiprotozoal drugs. Such small, stable, and cheap oil constituents, e.g., thymol, can serve as starting material to deliver new semi-synthetic antiparasitic agents with improved activity profiles.

## Figures and Tables

**Table 1:** The chemical composition of *Origanum onites* L. essential oil.

No	RRI ^a^	Compound	%	Identification Method ^b^
1	1018	Methyl 2-methyl-butyrate	0.1	MS
2	1024	Methyl 3-methyl-butyrate	tr ^c^	MS
3	1032	α-Pinene	0.5	RRI, MS
4	1035	α-Thujene	0.1	RRI, MS
5	1076	Camphene	0.2	RRI, MS
6	1118	β-Pinene	tr	RRI, MS
7	1159	δ-3-Carene	0.1	MS
8	1174	Myrcene	1.0	RRI, MS
9	1188	α-Terpinene	1.0	RRI, MS
10	1203	Limonene	0.2	RRI, MS
11	1213	1,8-Cineole	0.3	RRI, MS
12	1218	β-Phellandrene	0.2	RRI, MS
13	1246	(*Z*)- β-Ocimene	0.1	MS
14	1255	γ-Terpinene	2.1	RRI, MS
15	1280	*p*-Cymene	7.0	RRI, MS
16	1290	Terpinolene	0.1	RRI, MS
17	1393	3-Octanol	0.1	MS
18	1450	*trans*-Linalool oxide (*Furanoid*)	tr	MS
19	1452	α,*p*-Dimethylstyrene	tr	MS
20	1452	1-Octen-3-ol	0.3	MS
21	1474	*trans*-Sabinene hydrate	0.4	MS
22	1478	*cis*-Linalool oxide (*Furanoid*)	0.1	MS
23	1497	α-Copaene	tr	MS
24	1532	Camphor	tr	RRI, MS
25	1553	Linalool	9.7	RRI, MS
26	1571	*trans-p*-Menth-2-en-1-ol	tr	MS
27	1610	Calarene (=*β-gurjunene*)	tr	MS
28	1611	Terpinen-4-ol	0.7	RRI, MS
29	1612	β-Caryophyllene	0.7	RRI, MS
30	1620	Selina-5,11-diene	tr	MS
31	1628	Aromadendrene	0.2	MS
32	1638	*cis-p*-Menth-2-en-1-ol	tr	MS
33	1645	*cis*-Isodihydrocarvone	tr	MS
34	1661	Alloaromadendrene	tr	MS
35	1670	*trans*-Pinocarveol	tr	RRI, MS
36	1682	δ-Terpineol	tr	MS
37	1687	α-Humulene	tr	RRI, MS
38	1689	*trans*-Piperitol (=*trans-p-Menth-1-en-3-ol*)	tr	MS
39	1704	γ-Muurolene	tr	MS
40	1706	α-Terpineol	0.7	RRI, MS
41	1708	Ledene	0.1	MS
42	1719	Borneol	0.5	RRI, MS
43	1740	α-Muurolene	tr	MS
44	1751	Carvone	0.1	RRI, MS
45	1773	δ-Cadinene	0.1	MS
46	1776	γ-Cadinene	tr	MS
47	1798	Methyl salicylate	tr	RRI, MS
48	1845	*trans*-Carveol	0.1	MS
49	1864	*p*-Cymen-8-ol	0.1	RRI, MS
50	1940	4-Isopropyl salicylaldehyde	0.1	MS
51	1983	Piperitenone oxide	tr	RRI, MS
52	2008	Caryophyllene oxide	0.3	RRI, MS
53	2030	Methyl eugenol	tr	RRI, MS
54	2033	Epiglobulol	tr	MS
55	2050	(*E*)-Nerolidol	tr	MS
56	2071	Humulene epoxide-II	tr	MS
57	2098	Globulol	tr	MS
58	2104	Viridiflorol	tr	MS
59	2113	Cumin alcohol	tr	RRI, MS
60	2144	Spathulenol	0.1	MS
61	2181	Isothymol (=*2-Isopropyl-4-methyl phenol*)	tr	MS
62	2185	γ-Eudesmol	tr	MS
63	2186	Eugenol	tr	RRI, MS
64	2198	Thymol	1.8	RRI, MS
65	2221	Isocarvacrol (=*4-Isopropyl-2-methyl phenol*)	tr	MS
66	2239	Carvacrol	70.6	RRI, MS
67	2246	3-Isopropyl-2-methyl phenol	0.1	MS
68	2250	α-Eudesmol	tr	MS
69	2257	β-Eudesmol	tr	MS
70	2300	3-Isopropyl-5-methyl phenol	tr	MS
71	2392	Caryophylla-2(12),6-dien-5β-ol (=*Caryophyllenol II*)	tr	MS
		Monoterpene hydrocarbons	12.9	
		Oxygenated monoterpenes	84.9	
		Sesquiterpene hydrocarbons	1.1	
		Oxygenated sesquiterpenes	0.4	
		Diterpenes	0.6	
		Others	12.9	
		Total	99.9	

^a^ RRI: Relative retention indices calculated against *n*-alkanes; % calculated from FID data ^b^ Identification method based on the relative retention indices (RRI) of genuine standard compounds on the HP Innowax column; MS, identification was performed on the basis of computer matching of the mass spectra with those of the Wiley and MassFinder libraries and comparison with literature data. ^c^ tr, < 0.01.

**Table 2:** In vitro antiprotozoal activity and the cytotoxic potential of the *O. onites* essential oil and its components (IC_50_ values in μg/mL). Reference (standard) compounds: ^a^ melarsoprol, ^b^ benznidazole, ^c^ miltefosine, ^d^ chloroquine, ^e^ phodophyllotoxin.

Sample	*Trypanosoma brucei*	*Trypanosoma*	*Leishmania*	*Plasmodium*	Cytotoxicity
	*rhodesiense*	*cruzi*	*donovani*	*falciparum*	L6 cells
Standard compd	0.003 ^a^	0.44 ^b^	0.2 ^c^	0.056 ^d^	0.004 ^e^
*O. onites* Essential Oil	0.18 ± 0.004	>90	17.8 ± 3.7	7.9 ± 0.3	>90
Carvacrol	0.15 ± 0.04	>90	13.1 ± 3.9	6.4 ± 0.9	48.8 ± 1.1
Thymol	0.11 ± 0.01	>90	17.3 ± 4.1	5.7 ± 0.03	51.8 ± 6.2
α-Pinene	0.42 ± 0.24	>90	81.9 ± 9	10.7 ± 1.2	87.8 ± 3
Myrcene	22 ± 14.8	>90	48.2 ± 12.7	>20	>90
α-Terpinene	3.1 ± 1.6	49.1 ± 7.3	10.5 ± 1.7	3.7 ± 1.5	84.7 ± 4.3
γ-Terpinene	32.9 ± 26	>90	>90	>20	>90
*p*-Cymene	45.0 ± 27	>90	>90	>20	>90
*L*-Linalool	3.6 ± 2.5	>90	86.3 ± 2.8	>20	>90
Terpinen-4-ol	0.66 ± 0.4	46.8 ± 7.4	68.7 ± 14.2	>20	43.3 ± 16.5
β-Caryophyllene	28.9 ± 11.8	50.1 ± 12.5	52.4 ± 17.4	12.8	62.2 ± 7
α-Terpineol	0.56 ± 0.4	61.0 ± 2.1	75.9 ± 4.7	>20	32.3 ± 0.1
Borneol	24.3 ± 11.8	>90	52.1 ± 16.6	>20	>90
Carvacrol Methyl Ether	17.0 ± 22	>90	17.5 ± 1.7	>20	>90
Thymol Methyl Ether	4.1 ± 1.97	>90	86.0 ± 1.9	>20	>90
Thymoquinone	0.11 ± 0.02	18.2 ± 4.3	1.7 ± 0.16	2.6 ± 0.16	20.5 ± 8.1

The stock solutions were prepared by dissolving the essential oil and its components in 100% Dimethylsulfoxide at 10 mg/mL concentration and further diluted in assay medium (maximum DMSO concentration 1%). The IC_50_ values are means of two independent determinations ± deviation from the mean.

**Table 3:** In vivo activity of *O. onites oil* and its components against *T. brucei brucei* STIB795 mouse model.

Compound	Dosing Regimen (mg/kg) × 4 days	Injection	Cured/Infected	Mean Survival (days)
Essential oil	100	i.p.	0/4	5.25
Thymol	100	i.p.	0/4	9.0
Carvacrol	100	i.p.	0/4	5.25
Control ^a^	-	-	0/4	5.25
Standard ^b^	5.0	i.p.	4/4	>30

^a^ Untreated control to determine the mean survival. ^b^ Pentamidine.
